# Disability digital divide: survey of accessibility of eHealth services as perceived by people with and without impairment

**DOI:** 10.1186/s12889-023-15094-z

**Published:** 2023-01-27

**Authors:** Linda Pettersson, Stefan Johansson, Ingrid Demmelmaier, Catharina Gustavsson

**Affiliations:** 1 Center for Clinical Research Dalarna, Uppsala University, Nissers Väg 3, SE-791 82 Falun, Sweden; 2 Department of Public Health and Caring Sciences, Uppsala University, Box 564, SE-751 22 Uppsala, Sweden; 3 Primary Healthcare Center Mora, Mora Hospital, SE-792 85 Mora, Sweden; 4 School of Electrical Engineering and Computer Science, KTH Royal Institute of Technology, SE-100 44, Stockholm, Sweden; 5 School of Health and Welfare, Dalarna University, SE-791 88 Falun, Sweden

**Keywords:** eHealth, Impairment, Accessibility, Digital Inclusion, Universal design

## Abstract

**Background:**

Sustainable and effective eHealth requires accessibility for everyone. Little is known about how accessibility of eHealth is perceived among people with various impairments. The aim of this study was to compare use and perceived difficulty in the use of eHealth among people with and without impairment, and how different types of impairment were associated with perceived difficulty in the use of eHealth.

**Methods:**

This study used data collected in a nationwide survey in Sweden. Snowball sampling was used to recruit participants with self-reported impairment, from June to October 2019. In February 2020, the survey was posted to people in the general population who were matched to the participants with impairment by age, gender and county of residence. Multiple logistic regression was used to analyse the use of four eHealth services, and perceived difficulty in the use of six eHealth services.

**Results:**

In total, 1631 participants with, and 1084 participants without impairment responded to the survey. Participants with impairment reported less use and more difficulty in the use of all eHealth services as compared to participants without impairment. When comparing types of impairment, booking healthcare appointments online was least used and most avoided by participants with communication, language and calculation impairments (adjusted odds ratio (aOR) use 0.64, 95% confidence interval (95%CI) 0.49–0.83; aOR avoid 1.64, 95%CI 1.19–2.27), and intellectual impairments (aOR use 0.28, 95%CI 0.20–0.39; aOR avoid 2.88, 95%CI 1.86–4.45). The Swedish national web-portal for health information and services, 1177.se, was reported difficult to use the most among participants with communication, language and calculation impairments (aOR 2.24, 95%CI 1.50–3.36), deaf-blindness (aOR 11.24, 95%CI 3.49–36.23) and hearing impairment (aOR 2.50, 95%CI 1.17–5.35).

**Conclusions:**

The results confirm the existence of an eHealth disability digital divide. People with impairment were not one homogeneous group, but differed in perceived difficulties in regard to eHealth. Based on a purposeful subgrouping of impairments, we showed that people with communication, language and calculation impairments, and intellectual impairments, reported least use and most difficulty in using eHealth. The findings can guide further research in creating eHealth that is accessible for all, including those with the most significant difficulties.

**Supplementary Information:**

The online version contains supplementary material available at 10.1186/s12889-023-15094-z.

## Background

eHealth services can improve access to healthcare with limited resources [1–3]. In line with legislation [4] and human rights principles [5], and to ensure effective use of health services [6], eHealth services should be accessible for all. In the context of eHealth, accessibility refers to the extent to which all users can access and use the digital services to achieve an intended outcome [7]. However, when evaluated, many eHealth services are not accessible for people with impairment [8–11]. Barriers in the digital design can pose limitations on participation in eHealth for people with impairment and thereby cause disability [7, 12, 13]. It is known that eHealth users are characterised by being younger, more educated, richer, cohabitant, living in cities, having higher eHealth literacy and fewer chronic diagnoses compared to non-users [14, 15].

In Sweden, two national surveys by Statistics Sweden (SCB) and The Swedish Internet Foundation (IIS) continuously monitor internet use [16, 17], and also report data to the Eurostat reports on internet use [18]. However, these surveys have included only a small number of participants with impairment. If mentioned, people with impairments are presented as one homogeneous group, without investigating whether there are differences in internet use between types of impairment.

In a previous nationwide survey on internet use among people with impairments [19], we used snowball sampling instead of conventional survey methods, to attain participation from groups of people who had not been represented in the afore mentioned surveys. The results of our survey provided support for the existence of a disability digital divide in Sweden [19]. However, that survey did not explicitly target the use of eHealth services. Recently, it has been suggested that people with different types of impairment have different types and amounts of difficulty using the internet [19, 20]. Therefore, the aim of this study was to compare use and perceived difficulty in the use of eHealth among people with and without impairment, and how different types of impairment were associated with perceived difficulty in the use of eHealth.

## Methods

### Study design

This study had a cross-sectional comparative design and used data from the nationwide survey ‘Swedes with impairment and the Internet 2019’ (SMFOI19) which was distributed to people with impairment and then to matched individuals without impairment in the general population.

### Participants

People with self-reported impairment were recruited to the survey. They participated after having received the following information: ‘By impairment we mean such limitations that clearly affect how you live your life. Do you have such limitations?’. Then, people without impairment in the general population were invited to participate. For each participant with impairment, six matched individuals were invited to participate. Participants who received the survey as matched controls, but responded that they had an impairment, were reallocated and analysed as participants with impairment.

### Study procedures

A survey on internet use among people with impairment had been developed in 2017 to mirror two Swedish nationwide surveys on internet use [16, 17]. In 2019, the survey was further elaborated upon and questions on eHealth were added. The SMFOI19 survey questionnaire entailed 47 questions on various aspects of internet use as well as background characteristics including one question on type of impairment and diagnoses. The wording of the survey questions and response options were developed in close collaboration with members of the Begripsam group, whom all have lived experience of impairment. Several optional ways to respond to the survey were provided: by online or paper questionnaire, or by telephone or onsite interview. Reading support, interviews in sign language, complementary pictograms and support from a speech therapist was offered on request.

Snowball sampling was used from June to October 2019 to recruit participants with self-reported impairment to the SMFOI19 survey. Snowball sampling is a suitable sampling method to reach so called ‘rare populations’, which are hard to reach by conventional methods in population studies [21]. Information about the survey and recruitment of participants was distributed nationwide through social media and networks within disability organisations: home pages, e-mail contact lists, newsletters and by personal communication. The survey information endorsed everyone to share and redistribute the survey.

Then, in February 2020, the SMFOI19 survey was sent by post to people in the general population that were randomly selected to match the participants with impairment by age, gender and county of residence. Addresses of the matched individuals were provided from the Swedish state personal address register [22].

### Data collection

In the present study the survey questions in SMFOI19 that concerned use of eHealth, perceived difficulty in the use of eHealth, as well as impairments and diagnoses and other background characteristics, were analysed.

#### Dependent variables

Use of eHealth was measured by:

- The variable ‘Use of booking healthcare appointments online’ was combined from the questions: ‘Use of booking medical appointments online’ and ‘Use of booking dental appointments online’ with checkbox response options for reporting ‘I use’.
- The variable ‘Use of digital identification’ was combined from the questions: ‘Use of the digital identification app Mobile BankID’ and ‘Use of digital identification other than the app Mobile BankID’ with checkbox response options for reporting ‘I use’. Digital identification is a prerequisite to log in to most of the public eHealth services in Sweden, making it important to analyse in relation to the use of eHealth.

Difficulty in the use of eHealth was measured by:

- The variable ‘Booking healthcare appointments online’ was combined from the questions ‘Booking medical appointments online’ and ‘Booking dental appointments online’, with three response options: ‘If possible, I avoid booking appointments online’, ‘I try to book all my appointments online’ or ‘not applicable’. The response option ‘not applicable’ was excluded from the analysis.
- The variable ‘Digital identification’ was combined from the questions ‘The digital identification app Mobile BankID’ and ‘Digital identification other than the app Mobile BankID’, with three response options: ‘difficult to use’, ‘easy to use’ or ‘not applicable’. The response option ‘not applicable’ was excluded from the analysis.
- The website of the Swedish Social Insurance Agency (SSIA), with three response options: ‘difficult to use’, ‘easy to use’ or ‘not applicable’. The response option ‘not applicable’ was excluded from the analysis.
- The Swedish national web-portal for health information and eHealth services, 1177.se, with three response options: ‘difficult to use’, ‘easy to use’ or ‘not applicable’. The response option ‘not applicable’ was excluded from the analysis.

#### Independent variables

- Gender was analysed as ‘woman’ and ‘man’, whereas the response options ‘Other gender’ and ‘Prefer not to answer’ were excluded from the analysis.
- Age was divided into four categories: < 30, 30–44, 45–69 and ≥ 70 years.
- Impairment was measured by one question, followed by 43 checkbox response options on activity limitations and/or diagnoses and a free-text response option for providing information on other impairments (Additional file 1). The relevant exposure was set to be the type of impairment, as it has equal effect on the outcome disablement by design, regardless of whether the impairment is caused by a formal diagnosis (e.g. dyscalculia), another diagnosis with equal resulting impairment (e.g. aphasia) or an activity limitation without having received a formal diagnosis (e.g. perceived calculation impairment). The responses to the question on impairments were grouped by three of the authors, based on our competence in medicine, digital accessibility and human computer interaction, as well as empirical research [13] of similar co-morbidity and functioning, to a conceptual model of purposeful subgrouping of impairments. The three authors independently examined the activity limitations and diagnoses represented among the participants and categorised them into groups of impairments. Then, the authors compared the categorisations. There was almost complete interrater agreement. The very few disagreements were discussed until consensus of a final subgrouping of impairments was reached. Multiple responses were allowed in the reporting of impairments and diagnoses. Therefore, individuals could be included in more than one of the subgroups of impairments.

#### Background characteristics

Data was collected on participants’ educational level, occupation, income, professional support in everyday life, accommodation, and access to digital devices (computer, tablet and smart phone).

### Data analysis

Pair-wise deletion was used when responses were missing on individual items. Multiple logistic regression models were built by a linear model with robust (Huber-White) standard errors and a direct approach in entering empirically based relevant independent variables [23]. The association between independent variables and the outcomes were adjusted for background characteristics with confounding potential (gender and age) [14, 24]. Multicollinearity was assessed in relation to a predetermined cut-off. To assess the robustness of the parameter estimates, models were fitted with and without independent variables with wide confidence intervals. A *p*-value ≤ 0.05 was accepted as statistically significant. Data was analysed using the statistical software IBM SPSS 26.0 [25] and Microsoft Excel.

## Results

**Fig. 1:**
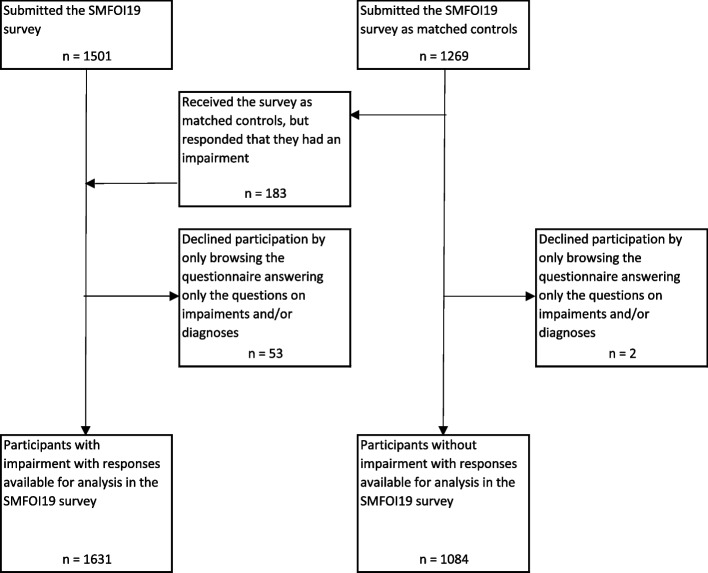
Flowchart of participation in the survey SMFOI19: ‘Swedes with impairment and the Internet 2019’

A flowchart of participation is reported in Fig. 1. Fifty-three participants with impairment and two participants without impairment withdrew from the survey after responding only to the question on impairments and diagnoses. One-hundred and eighty-three participants (14%) received the survey as matched controls, but responded that they had impairment and were reallocated to the group of participants with impairment.

Background characteristics for participants with impairment (*n* = 1631) and participants without impairment (*n* = 1084) are displayed in Table 1. Participants with impairment had lower income and less access to digital devices, as compared to participants without impairment. Also, more participants with impairment had education in special education schools and professional support in everyday life.

**Table 1:** Background characteristics of participants with and without impairment

	Participants with impairment	Participants without impairment
	*n* = 1631	*n* = 1084
	n(%)	n(%)
Gender	*n* = 1356	*n* = 1060
Women	937(69)	780(74)
Men	419(31)	280(26)
Age	*n* = 1388	*n* = 1069
< 30 years	176(13)	58(5)
30–44 years	304(22)	156(15)
45–69 years	773(56)	716(67)
≥ 70 years	135(10)	139(13)
Education	*n* = 1339	*n* = 1040
Compulsory school	134(10)	74(7)
Special education school	152(11)	2(0)
Upper secondary school, Vocational school or Folk high school	567(42)	442(43)
University	486(36)	522(50)
Occupation	*n* = 1367	*n* = 1060
Retired	273(17)	304(28)
Outside of the labour market (Disability related early retirement, Daily activity centre or Temporary disability allowance)	519(38)	18(2)
Working	492(30)	745(69)
Student	136(8)	36(3)
Not working (Unemployed, Parental-leave or Sick-leave)	132(10)	22(2)
Monthly income	*n* = 1157	*n* = 866
< 5000 SEK	53(5)	10(1)
5000–24 999 SEK	714(62)	228(26)
≥ 25 000 SEK	390(34)	628(73)
Professional support in everyday life	*n* = 1381	*n* = 1063
Have professional support in everyday life	497(36)	6(1)
Home based support by municipal care services	112(8)	2(0)
Personal assistants	90(7)	0(0)
Supported-Living staff, support persons or similar	214(15)	0(0)
Trustee	111(8)	0(0)
Relative	81(6)	4(0)
Other support	54(4)	4(0)
No support	884(64)	1057(99)
Accommodation	*n* = 1365	*n* = 1059
Supported accommodation	104(8)	1(0)
Group living	52(4)	1(0)
Service apartment	43(3)	0(0)
Other supported accommodation	9(1)	0(0)
Rental apartment, Condominium or House	1261(92)	1058(100)
Rental apartment	470(34)	168(16)
Condominium	285(21)	239(22)
House	506(37)	651(61)
Access to digital devices	*n* = 1456	*n* = 1067
Lack access to computer or portable device	241(17)	93(9)
No device	29(2)	7(1)
Only computer at home	75(5)	24(2)
Only smart phone	78(5)	19(2)
Only tablet	17(1)	1(0)
Smart phone and tablet	42(3)	39(4)
Have access to computer and portable device	1215(83)	974(91)
Computer and smart phone	418(29)	264(25)
Computer and tablet	65(4)	25(2)
Computer, smart phone and tablet	732(51)	688(64)
Number of reported impairments	*n* = 1631	*n* = 1084
1	590(36)	0(0)
2	241(15)	0(0)
3	168(10)	0(0)
4	114(7)	0(0)
5	107(7)	0(0)
6	92(6)	0(0)
≥ 7	319(20)	0(0)

**Fig. 2:**
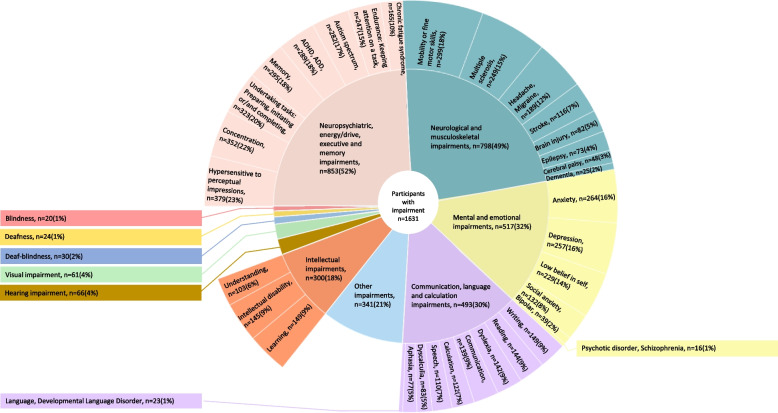
Impairments (outer circle) purposefully subgrouped (inner circle). Reported as number of participants (n) and proportion of all participants with impairment (%). ‘Limitations that clearly affect how you live your life’, but without having received a diagnosis could also be reported, e.g. perceived Calculation difficulty without the diagnosis Dyscalculia. The proportions add up to over 100% as multiple responses were allowed. The following response options were separate in the questionnaire (Additional file 1), but grouped in this figure: ADHD + ADD, Deafness, childhood onset + Deafness, acquired in adulthood, Dementia + Parkinson Disease, Mobility impairment, difficulties in fine motor skills + Difficulties to sit

In total, 6728 impairments and diagnoses were reported by the 1631 participants (Fig. 2). Multiple impairments were reported by 64% (*n* = 1041) (Table 1). Impairments were distributed into the following subgroups: neuropsychiatric, energy/drive, executive and memory impairments (*n* = 853), neurological and musculoskeletal impairments (*n* = 798), mental and emotional impairments (*n* = 517), communication, language and calculation impairments (*n* = 493), other impairments specified in free-text (*n* = 341), intellectual impairments (*n* = 300), hearing impairment (*n* = 66), visual impairment (*n* = 61), deaf-blindness (*n* = 30), deafness (*n* = 24) and blindness (*n* = 20).

### Use of eHealth

**Fig. 3:**
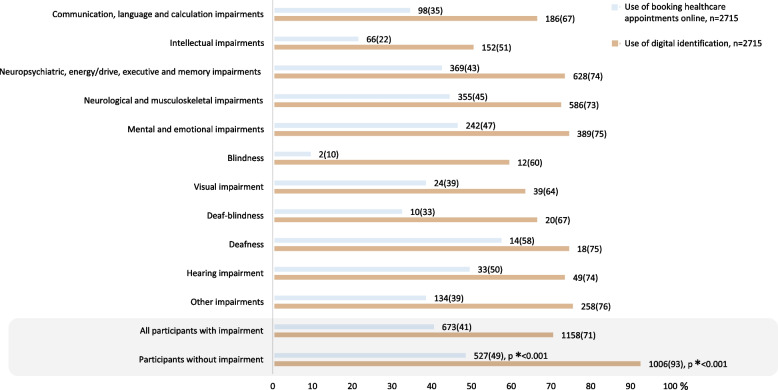
Proportions of participants reporting use of eHealth. * Chi2-test 2-sided *p*-value comparing participants with impairment to participants without. Bar sizes are percentages; numbers next to the bars are number and proportion, n(%)

**Fig. 4:**
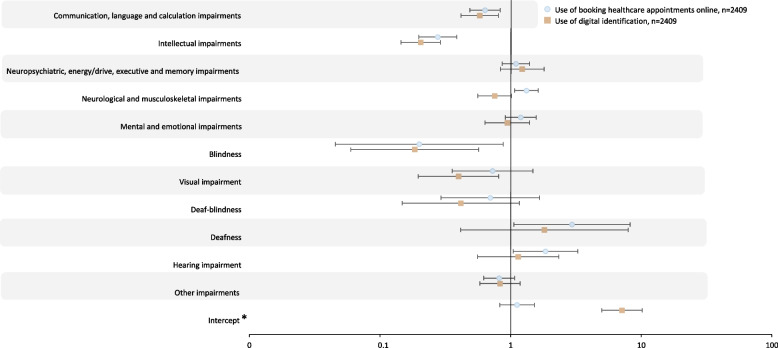
Multiple logistic regression modelling use of eHealth, adjusted odds ratios. * Reference group is participants without impairment, adjusted for age (reference below 30 years old) and gender (reference female). Numbers behind the figure are outlined in Additional file 2

Booking appointments online was used less among participants with impairment (41% *n* = 673) than without impairment (49% *n* = 527) (Fig. 3). Booking appointments online was used the least among participants with communication, language and calculation impairments (aOR 0.64, 95%CI 0.49–0.83), intellectual impairments (aOR 0.28, 95%CI 0.20–0.39) and blindness (aOR 0.20, 95%CI 0.05–0.88, *n* = 2/10%) (Fig. 4). More likely to report use of booking appointments online were participants with neurological and musculoskeletal impairments (aOR 1.32, 95%CI 1.08–1.63), deafness (aOR 2.95, 95%CI 1.06–8.22) and hearing impairment (aOR 1.85, 95%CI 1.05–3.27). Not using digital identification was more common among participants with impairment (29% *n* = 473) compared to participants without impairment (7% *n* = 78) (Fig. 3). Digital identification was used the least among participants with communication, language and calculation impairments (aOR 0.58, 95%CI 0.42–0.81), intellectual impairments (aOR 0.21, 95%CI 0.15–0.29), blindness (aOR 0.19, 95%CI 0.06–0.57) and visual impairment (aOR 0.40, 95%CI 0.20–0.81) (Fig. 4).

### Difficulty in the use of eHealth

**Fig. 5:**
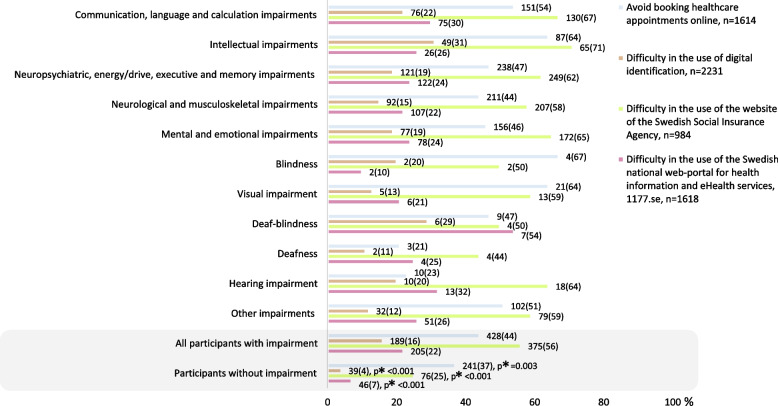
Proportions of participants reporting difficulty in the use of eHealth. * Chi2-test 2-sided *p*-value comparing participants with impairment to participants without. Bar sizes are percentages; numbers next to the bars are number and proportion, n(%)

**Fig. 6:**
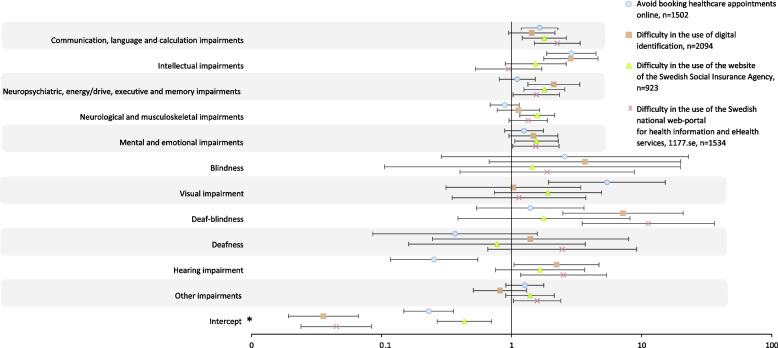
Multiple logistic regression modelling difficulty in the use of eHealth, adjusted odds ratios. * Reference group is participants without impairment, adjusted for age (reference below 30 years old) and gender (reference female). Models were fitted with and without independent variables with wide confidence intervals to assess the robustness of the parameter estimates. Numbers behind the figure are outlined in Additional file 3

Participants with impairment reported more difficulty in the use of all eHealth services compared to participants without impairment (Fig. 5). All subgroups of impairments reported more difficulty in the use of eHealth, with the one exception of participants with hearing impairment on the variable – booking appointments online (aOR 0.25, 95%CI 0.12–0.55) (Fig. 6).Booking appointments online was avoided by 44% (*n* = 428) of the participants with impairment and 37% (*n* = 241) of participants without impairment (Fig. 5). Booking appointments online was avoided the most among participants with communication, language and calculation impairments (aOR 1.64, 95%CI 1.19–2.27), intellectual impairments (aOR 2.88, 95%CI 1.86–4.45) and visual impairment (aOR 5.40, 95%CI 1.92–15.18) (Fig. 6).

Among those reporting an opinion about digital identification, it was reported as difficult to use by 16% (*n* = 189) of participants with impairment and by 4% (*n* = 39) of participants without impairment (Fig. 5). Use of digital identification was reported as difficult to use the most among participants with intellectual impairments (aOR 2.86, 95%CI 1.77–4.62) and deaf-blindness (aOR 7.18, 95%CI 2.47–20.86, *n* = 6/29%) (Fig. 6).

The SSIA website received the largest reporting of difficulty in use, more so among participants with impairment (56% *n* = 375), as compared to participants without impairment (25% *n* = 76) (Fig. 5). The SSIA website was reported as difficult to use the most among participants with communication, language and calculation impairments (aOR 1.79, 95%CI 1.21–2.64), neuropsychiatric, energy/drive, executive and memory impairments (aOR 1.79, 95%CI 1.25–2.56), neurological and musculoskeletal impairments (aOR 1.57, 95%CI 1.16–2.14) and mental and emotional impairments (aOR 1.55, 95%CI 1.06–2.29) (Fig. 6).

Among those reporting an opinion on the 1177.se web-portal, 22% (*n* = 205) of participants with impairment and 7% (*n* = 46) of participants without impairment reported that it was difficult to use (Fig. 5). The 1177.se web-portal was reported as difficult to use the most among participants with communication, language and calculation impairments (aOR 2.24, 95%CI 1.50–3.36), deaf-blindness (aOR 11.24, 95%CI 3.49–36.23, *n* = 7/54%) and hearing impairment (aOR 2.50, 95%CI 1.17–5.35) (Fig. 6).

## Discussion

This study showed an eHealth disability digital divide in that participants with impairment reported less use and more difficulty in the use of eHealth compared to participants without impairment. When subgrouping impairments, which to our knowledge has not been previously done in this detailed way, the least use and most difficulty using eHealth were shown among participants with communication, language and calculation impairments, and intellectual impairments.

In line with our results, other studies have shown that people with impairment use eHealth services less [11] and report more difficulties [9] than people without impairment. The one study that did not show less use of the internet for health-related activities, did not include people with such impairments, who in the present study had the least use of, and the most difficulty in using eHealth, i.e. communication, language and calculation impairments, and intellectual impairments [26].

Booking healthcare appointments online and the SSIA website were the most difficult eHealth service in this study. Using these services is complex, as they require digital identification to log in, require understanding of digital functions and features and require considerable executive functions to operate. Twice as many participants with impairment avoided booking healthcare appointments online and reported difficulty in using the SSIA website, as compared to participants without impairment.

Among participants with communication, language and calculation impairments, intellectual impairments and all visual impairments (visual impairment, blindness and deaf-blindness), most of the eHealth services were reported as difficult to use: i.e., digital identification, the 1177.se web-portal and booking healthcare appointments online.

Participants with impairments relating to communication, language and calculation (Fig. 2) were grouped together based on similar co-morbidity and functioning regarding working memory, symbol interpretation and comprehension [27–29]. Indeed, the results confirmed that they presented a similar pattern of less use and more difficulties in the use of eHealth. Previous literature has discussed the limitation of eHealth lacking non-verbal communication (such as eye-contact) and communicative emotion (such as vocal intonation) [30], and concerns have been raised about the patient—care provider relationship and therapeutic alliance of sporadic contacts [31], which could plausibly be important among people with communication, language and calculation impairments. Design features to increase their accessibility are standards for understandable texts, text-to-speech technology, audio, speech-to-text technology and the possibility to import numbers from a reliable source rather than to enter them manually [32], as well as visualisations [33] and allowing for longer duration of communication [34].

The least use and most frequent difficulties in use of eHealth overall in this study were reported among participants with intellectual impairments. This is consistent with findings in other studies showing that people with intellectual impairments struggle with using the web [35, 36]. Improvements in the digital design that increase accessibility in relation to intellectual impairments are plain language with short sentences, visualisations, clear icons, calm background, audio including narrative information, intuitive navigation and language options [35, 37], whereas disablement by design can be caused by updates requiring new learning [9].

Participants with visual impairment reported less use of digital identification and more frequently avoiding booking healthcare appointments online, as compared to all other participants. Previous studies showed that not complying with design guidelines made digital services visually inaccessible, and that accessibility improved after re-design [38, 39]. People with progressive visual impairment can usually appreciate assistive devices such as magnifiers to reinforce sight [40]. Assistive device compatibility could aid their access to eHealth [41], but also technical options for magnifying, such as contrast and brightness [42]. Childhood onset severe visual impairment, on the other hand, makes communicative development rely on senses other than vision. Among participants with blindness, few reported use of digital identification or booking healthcare appointments online. Text-to-speech technology and audio could increase accessibility of eHealth [32, 42]. However, people with blindness often use their own assistive device with e.g. functions for screen reading, since built-in audio functions on webpages may need visual functioning to initiate. Thus, assistive device compatibility, in the 1177.se web-portal and for digital identification, is important. In the present study, participants with deaf-blindness had the most difficulty using digital identification and the 1177.se web-portal, as compared to all other participants. The diagnosis deaf-blindness defines when the level of visual and/or hearing impairment is too severe for one to compensate for the other. When people with deaf-blindness have residual hearing or vision, the same design functions as for other sensory impairments can make eHealth accessible for them. Otherwise, the tactile sense is important for their communication and eHealth use [9, 43].

In the present study, the only exception to the disability digital divide was booking healthcare appointments online, which was used by a higher proportion of participants with neurological and musculoskeletal impairments, deafness, and hearing impairment, as compared to participants without impairment. Also, participants with hearing impairment alongside participants without impairment were least likely to avoid booking healthcare appointments online. This finding is reasonable, since the telephone is difficult with hearing impairment and written information or administrative procedures can be a secure option avoiding potential loss of spoken communication [44]. Therefore, eHealth may be an important tool for improved overall healthcare accessibility for people with hearing loss.

Our results show that people with impairment have more difficulties using eHealth than people without impairment. At the same time, people with impairment are under-represented in eHealth research [45]. The results in the present study demonstrated that people with similar functional impairments report similar use of eHealth and difficulties using eHealth. Henni et al. recently reported the same findings in a scoping review, by combining data from multiple studies [9]. Our analyses showed that the overlaps due to participants reporting several impairments did not exceed the predetermined cut-off, and thus people with multi-morbidity could be represented in the results. Hence, the purposeful subgrouping of impairments can be a useful tool to understand who will probably perceive difficulties in the use of eHealth. This can be used to inform designers and policy makers as to who should be involved in the design process of eHealth services. Our subgrouping of impairments shows that it is especially important that people with communication, language and calculation impairments, and intellectual impairments, are involved in the design of eHealth, as they reported the least use and the most difficulties. Evaluations show that many eHealth services do not comply with accessibility standards [8, 10]. Further, there is a critique that accessibility standards are too narrow, lacking cognitive accessibility [9, 34, 35], which was shown to be a prominent aspect for the impairments of the participants who in this study reported the most difficulties in the use of eHealth services. We suggest that including people with these types of impairment in co-design processes when developing eHealth services, would increase the focus on cognitive accessibility and complement existing accessibility standards. The importance of user participation in eHealth development is acknowledged by both research and policy makers, as it can improve accessibility [46–49]. Co-design of eHealth by user participation involving people with the most significant difficulties, will thereby produce eHealth services that are usable and accessible to the widest range of people, i.e. universal design [7, 9]. In short, designing for people with the most difficulties will produce eHealth for the whole population. Our subgrouping of impairments can also be used when evaluating effects on eHealth investments. If large proportions of the population avoid eHealth services, the favourable effects of those services will be lower than anticipated [6]. Thus, the use of eHealth among people with impairment is important for accurately measuring the effects of eHealth and for obtaining maximal gain on eHealth investments.

### Strengths and limitations

It is a major strength of this study that, by use of the snowball sampling method, we managed to achieve substantial participation of people with impairment, i.e. among populations considered hard-to-reach by conventional survey sampling methods [21]. However, the use of snowball sampling mainly through online survey, plausibly reached more digitally literate people, which limits the generalisation of the findings to all people with impairment in Sweden. In addition, previous research has shown that self-assessment of digital literacy, might result in underestimation of difficulties [50, 51]. In summary, our results succeeded in measuring the disability digital divide of eHealth, but plausibly underestimated its severity.

A strength of this study is that we showed differences not only between people with and without impairment, but also differences between subgroups of impairments. We believe it to be a rigorous strategy to survey all significant impairments, if managing methodological challenges in the statistical analysis. Since having multiple impairments was more common than having a single diagnosis, the risk of misclassification bias in selecting a primary impairment would be substantial. Multicollinearity did not exceed the predetermined cut-off, sensitivity analyses did not have significant impact on odds ratios and outcomes were not associated with number of reported impairments. This contributed to a differentiated knowledge of the heterogeneity in use and difficulty in use of eHealth between purposefully grouped impairments. The proposed subgrouping of impairments is a first attempt that will need to be validated in future studies.

It is notable that 14% of those in the general population who responded to the survey reported having impairment. This roughly corresponds to estimates of prevalence of impairment in the population [52]. It indicates that how we constructed the question on impairment was successful in attaining appropriate information of impairment in the general population. There are multiple approaches to constructing questions on impairment, which should be guided by the purpose, e.g. in medicine or for legal definitions [13]. We want to stress the importance of asking about impairments in relation to the outcome. Functioning is indeed continuous, not dichotomous, and differs depending on the activity and the context. We asked participants to report impairment that ‘clearly affect how you live your life’ since eHealth usage requires high level of functioning. We developed the question on impairment in collaboration with people having different types of impairment, which strengthens the validity of the question. Altogether, by this approach we believe we present results that are more credible as compared to other national surveys which have used generic questions, without specifying type of impairment [16, 17]. We suggest that the question used in this study is favourable for reporting impairment and should also be used in other studies of accessibility of eHealth.

The body of literature is still scarce on accessibility of eHealth. However, there are more studies on digital accessibility in general [11, 19, 35–38, 43]. We find it reasonable to discuss our findings in relation to studies of web accessibility and digital technology in general. However, eHealth involves complex services and the interplay with health literacy compels caution in comparability. Further, there might be other factors not investigated in this study that are associated with accessibility of eHealth, for example eHealth literacy [6, 53] and socioeconomic factors [14, 15]. This study was undertaken prior to the COVID-19 pandemic. The pandemic has forced community services online [34] and it is plausible that digital participation has changed in the population, which raises questions on whether the findings are valid in a post-pandemic context. We are currently undertaking a survey to investigate changes in the disability digital divide related to the COVID-19 pandemic.

## Conclusions

The result confirmed an eHealth disability digital divide in that participants with impairment reported less use and more difficulty in the use of eHealth compared to participants without impairment. It also extended our knowledge that people with impairment are not one homogeneous group, but differ in perceived difficulties in regard to eHealth. Based on a purposeful subgrouping of impairments, we showed that people with communication, language and calculation impairments, and intellectual impairments, reported the least use and most difficulty in using eHealth. The shown diversity in the disability digital divide and the purposeful grouping of impairments can guide researchers and designers in developing eHealth that is accessible for all people, including those with the most difficulties.

## Supplementary Information

**Additional file 1.** Questions about impairments.

**Additional file 2.** Multiple logistic regression modelling use of eHealth. Unadjusted and adjusted model per dependent variable.

**Additional file 3.** Multiple logistic regression modelling difficulty in the use of eHealth. Unadjusted and adjusted model per dependent variable.

## Data Availability

The datasets used and analysed during the current study are available from the corresponding author on reasonable request.

## Abbreviations

95%CI: 95% Confidence interval
aOR: Adjusted odds ratio
SMFOI19: Swedes with impairment and the Internet 2019
SSIA: The Swedish Social Insurance Agency
