# Supplementary material for: Disability digital divide: survey of accessibility of eHealth services as perceived by people with and without impairment
Source: BMC Public Health. 2023 Jan 27;23:181. doi: 10.1186/s12889-023-15094-z (PMC9880913; doi:10.1186/s12889-023-15094-z)
Supplement: Supplementary file 1 — Additional file 1. Questions about impairments. [file 12889_2023_15094_MOESM1_ESM.docx]

# Questions about diagnoses, impairments and difficulties

# Do you have an impairment?

By impairment, we mean such limitations that clearly affect how you live your life. Do you have such limitations?

- Yes
- No
- I do not know

**If you answered No or I do not know:** Move forward to page 4 in the survey ‘Questions about using the Web/access the Internet’

**If you answered Yes:** Please respond to this next question:

# Which of these diagnoses, impairments or difficulties do you have?

Go through the checklist and tic the boxes that apply to you. You can select several options. You can also tic the box ‘Other’ at the bottom of the list and specify other diagnoses, impairments or difficulties that are relevant to you.

- ADD
- ADHD
- Aphasia
- Autism Spectrum (Autism, Asperger Syndrome)
- Bipolar disorder
- Blindness
- Cerebral Palsy / CP
- Dementia, Alzheimer etc.
- Depression
- Dyslexia
- Dyscalculia
- Deafness, childhood onset
- Deafness, acquired in adulthood
- Deaf blindness
- Epilepsy
- Severe hearing impairment
- Severe visual impairment
- Acquired brain injury
- Headache, Migraine
- Communication difficulties
- Concentration difficulties
- Chronic Fatigue Syndrome / ME
- Hypersensitive to strong/sudden perceptual impressions (flickering lights, sudden or loud sounds, etc.)
- Reading difficulties
- Memory difficulties
- Multiple Sclerosis / MS
- Parkinson Disease
- Mathematic difficulties
- Mobility impairment, difficulties in fine motor skills
- Stroke
- Schizophrenia, psychotic disorder
- Low self-esteem / low belief in self
- Writing difficulties
- Social anxiety
- Language disorder, DLD
- Difficulties understanding
- Difficulties learning new things
- Difficulties getting started, or completing, tasks or activities
- Difficulties to sit
- Difficulties keeping attention on a task or activity
- Speech difficulties
- Intellectual impairment
- Anxiety
- Other, please describe:
