# Supplementary material for: Disability digital divide: survey of accessibility of eHealth services as perceived by people with and without impairment
Source: BMC Public Health. 2023 Jan 27;23:181. doi: 10.1186/s12889-023-15094-z (PMC9880913; doi:10.1186/s12889-023-15094-z)
Supplement: Supplementary file 2 — Additional file 2. Multiple logistic regression modelling use of eHealth. Unadjusted and adjusted model per dependent variable. [file 12889_2023_15094_MOESM2_ESM.docx]

### **Additional file 2.** Multiple logistic regression modelling use of eHealth. Unadjusted and adjusted model per dependent variable.

|  | Use of booking  healthcare appointments online | | | Use of  digital identification | | |
| --- | --- | --- | --- | --- | --- | --- |
|  |  | n=2715 | n=2409 |  | n=2715 | n=2409 |
| Independent variables | n(%) | OR(p-value) 95%CI | aOR(p-value) 95%CI | n(%) | OR(p-value) 95%CI | aOR(p-value) 95%CI |
| Communication, language and calculation impairments | 98(35) | 0.71**(0.003)** 0.56-0.89 | 0.64**(0.001)** 0.49-0.83 | 186(67) | 0.66**(0.003)** 0.50-0.87 | 0.58**(0.001)** 0.42-0.81 |
| Intellectual impairments | 66(22) | 0.31**(<0.001)** 0.23-0.42 | 0.28**(<0.001)** 0.20-0.39 | 152(51) | 0.25**(<0.001)** 0.19-0.34 | 0.21**(<0.001)** 0.15-0.29 |
| Attention, energy, executive and memory impairments | 369(43) | 1.07(0.52) 0.87-1.32 | 1.10(0.45) 0.86-1.40 | 628(74) | 0.96(0.80) 0.72-1.30 | 1.23(0.30) 0.84-1.80 |
| Neurological and musculoskeletal impairments | 355(45) | 1.18(0.08) 0.98-1.41 | 1.32**(0.008)** 1.08-1.63 | 586(73) | 0.80(0.07) 0.63-1.02 | 0.75(0.06) 0.56-1.02 |
| Mental and emotional impairments | 242(47) | 1.43**(0.004)** 1.12-1.81 | 1.20(0.20) 0.91-1.57 | 389(75) | 1.16(0.37) 0.84-1.58 | 0.94(0.772) 0.64-1.40 |
| Sensory impairments |  |  |  |  |  |  |
| *Blindness* | 2(10) | 0.12**(0.005)** 0.027-0.53 | 0.20**(0.03)** 0.05-0.88 | 12(60) | 0.28**(0.006)** 0.12-0.69 | 0.19**(0.003)** 0.06-0.57 |
| *Visual impairment* | 24(39) | 0.77(0.36) 0.44-1.34 | 0.73(0.38) 0.36-1.48 | 39(64) | 0.49**(0.02)** 0.27-0.91 | 0.40**(0.01)** 0.20-0.81 |
| *Deaf-blindness* | 10(33) | 0.61(0.21) 0.28-1.33 | 0.70(0.42) 0.29-1.67 | 20(67) | 0.42**(0.03)** 0.19-0.91 | 0.42(0.096) 0.15-1.17 |
| *Deafness* | 14(58) | 1.81(0.18) 0.76-4.35 | 2.95**(0.04)** 1.06-8.22 | 18(75) | 0.73(0.55) 0.26-2.03 | 1.81(0.43) 0.42-7.95 |
| *Hearing impairment* | 33(50) | 1.36(0.22) 0.83-2.24 | 1.85**(0.03)** 1.05-3.27 | 49(74) | 0.81(0.49) 0.45-1.47 | 1.14(0.72) 0.56-2.34 |
| Other impairments | 134(39) | 0.80(0.06) 0.63-1.01 | 0.82(0.15) 0.62-1.07 | 258(76) | 0.90(0.49) 0.67-1.22 | 0.83(0.30) 0.58-1.18 |
| Intercept * | 527(49) | 0.85**(0.003)** 0.77-0.95 | 1.12 (0.46) 0.83-1.52 | 1006(93) | 5.98**(<0.001)** 5.26-6.79 | 7.12**(<0.001)** 5.00-10.17 |

* *Reference group is participants without impairment, adjusted for age (reference below 30 years old) and gender (reference female)*. *95%CI: 95% confidence interval; aOR: odds ratio after adjusting for gender and age; statistically significant p-values are shown in bold.*
