# Supplementary material for: Disability digital divide: survey of accessibility of eHealth services as perceived by people with and without impairment
Source: BMC Public Health. 2023 Jan 27;23:181. doi: 10.1186/s12889-023-15094-z (PMC9880913; doi:10.1186/s12889-023-15094-z)
Supplement: Supplementary file 3 — Additional file 3. Multiple logistic regression modelling difficulty in the use of eHealth. Unadjusted and adjusted model per dependent variable. [file 12889_2023_15094_MOESM3_ESM.docx]

### **Additional file 3.** Multiple logistic regression modelling difficulty in the use of eHealth. Unadjusted and adjusted model per dependent variable.

|  | Avoid booking  healthcare appointments online | | | Difficulty in the use of  digital identification | | | Difficulty in the use of  the website of  the Swedish Social Insurance Agency | | | Difficulty in the use of  the Swedish national web-portal for health information and eHealth services, 1177.se | | |
| --- | --- | --- | --- | --- | --- | --- | --- | --- | --- | --- | --- | --- |
|  |  | n=1614 | n=1502 |  | n=2231 | n=2094 |  | n=984 | n=923 |  | n=1618 | n=1534 |
| Independent variables | n(%) | OR(p-value) 95%CI | aOR(p-value) 95%CI | n(%) | OR(p-value) 95%CI | aOR(p-value) 95%CI | n(%) | OR(p-value) 95%CI | aOR(p-value) 95%CI | n(%) | OR(p-value) 95%CI | aOR(p-value) 95%CI |
| Communication, language and calculation impairments | 151(54) | 1.60**(0.002)** 1.19-2.14 | 1.64**(0.003)** 1.19-2.27 | 76(22) | 1.71**(0.005)** 1.17-2.49 | 1.43(0.09) 0.95-2.16 | 130(67) | 1.79**(0.002)** 1.23-2.59 | 1.79**(0.004)** 1.21-2.64 | 75(30) | 2.13**(<0.001)** 1.46-3.12 | 2.24**(<0.001)** 1.50-3.36 |
| Intellectual impairments | 87(64) | 2.26**(<0.001)** 1.51-3.38 | 2.88**(<0.001)** 1.86-4.45 | 49(31) | 2.45**(<0.001)** 1.56-3.85 | 2.86**(<0.001)** 1.77-4.62 | 65(71) | 1.52(0.11) 0.91-2.53 | 1.53(0.12) 0.90-2.63 | 26(26) | 0.88(0.65) 0.51-1.52 | 0.95(0.87) 0.53-1.71 |
| Attention, energy, executive and memory impairments | 238(47) | 1.0(0.95) 0.76-1.35 | 1.10(0.54) 0.80-1.52 | 121(19) | 1.90**(0.002)** 1.25-2.87 | 2.11**(0.001)** 1.33-3.34 | 249(62) | 1.76**(0.001)** 1.25-2.47 | 1.79**(0.002)** 1.25-2.56 | 122(24) | 1.55**(0.02)** 1.06-2.27 | 1.55**(0.04)** 1.03-2.34 |
| Neurological and musculoskeletal impairments | 211(44) | 1.0(0.99) 0.79-1.26 | 0.88(0.35) 0.68-1.14 | 92(15) | 1.16(0.410) 0.82-1.63 | 1.13(0.53) 0.78-1.64) | 207(58) | 1.55**(0.003)** 1.16-2.08 | 1.57**(0.004)** 1.16-2.14 | 107(22) | 1.45**(0.02)** 1.05-1.99 | 1.34(0.09) 0.95-1.87 |
| Mental and emotional impairments | 156(46) | 0.96(0.79) 0.70-1.30 | 1.25(0.21) 0.88-1.76 | 77(19) | 1.25(0.28) 0.84-1.86 | 1.47(0.08) 0.96-2.27 | 172(65) | 1.62**(0.01)** 1.123-2.32 | 1.55**(0.03)** 1.06-2.29 | 78(24) | 1.14(0.49) 0.78-1.67 | 1.54**(0.04)** 1.019-2.316 |
| Sensory impairments |  |  |  |  |  |  |  |  |  |  |  |  |
| *Blindness* | 4(67) | 3.45(0.16) 0.62-19.01 | 2.57(0.40) 0.29-22.83 | 2(20) | 3.51(0.13) 0.68-18.13 | 3.66(0.13) 0.67-19.94 | 2(50) | 1.29(0.85) 0.09-18.70 | 1.44(0.78) 0.11-19.82 | 2(10) | 2.05(0.33) 0.49-8.62 | 1.88(0.42) 0.40-8.78 |
| *Visual impairment* | 21(64) | 3.59**(0.001)** 1.64-7.85 | 5.40**(0.001)** 1.92-15.18 | 5(13) | 0.99(0.99) 0.33-3.00 | 1.03(0.96) 0.31-3.40 | 13(59) | 1.93(0.15) 0.79-4.72 | 1.90(0.18) 0.74-4.92 | 6(21) | 1.25(0.67) 0.45-3.50 | 1.14(0.83) 0.35-3.72 |
| *Deaf-blindness* | 9(47) | 1.35(0.52) 0.54-3.40 | 1.39(0.50) 0.54-3.60 | 6(29) | 5.01**(0.002)** 1.83-13.71 | 7.18**(<0.001)** 2.47-20.86 | 4(50) | 1.67(0.51) 0.37-7.65 | 1.77(0.46) 0.39-8.14 | 7(54) | 9.17**(<0.001)** 2.82-29.81 | 11.24**(<0.001)** 3.49-36.23 |
| *Deafness* | 3(21) | 0.28(0.06) 0.07-1.07 | 0.37(0.18) 0.09-1.58 | 2(11) | 1.28(0.77) 0.24-6.71 | 1.40(0.71) 0.25-7.91 | 4(44) | 1.04(0.96) 0.25-4.33 | 0.77(0.74) 0.16-3.68 | 4(25) | 2.24(0.21) 0.63-7.92 | 2.45(0.18) 0.66-9.13 |
| *Hearing impairment* | 10(23) | 0.30**(0.001)** 0.15-0.62 | 0.25**(0.001)** 0.12-0.55 | 10(20) | 2.25**(0.03)** 1.10-4.62 | 2.21**(0.04)** 1.04-4.70 | 18(64) | 1.71(0.17) 0.79-3.70 | 1.66(0.21) 0.75-3.64 | 13(32) | 2.68**(0.01)** 1.25-5.74 | 2.50**(0.02)** 1.17-5.35 |
| Other impairments | 102(51) | 1.46**(0.02)** 1.07-1.99 | 1.26(0.17) 0.90-1.77 | 32(12) | 0.85(0.47) 0.54-1.33 | 0.81(0.39) 0.51-1.30 | 79(59) | 1.34(0.15) 0.90-2.01 | 1.38(0.14) 0.90-2.12 | 51(26) | 1.64**(0.01)** 1.11-2.43 | 1.57**(0.03)** 1.03-2.39 |
| Intercept * | 241(37) | 0.59**(<0.001)** 0.51-0.67 | 0.23**(<0.001)** 0.15-0.36 | 39(4) | 0.06**(<0.001)** 0.05-0.08 | 0.04**(<0.001)** 0.02-0.07 | 76(25) | 0.40**(<0.001)** 0.33-0.49 | 0.43**(0.001)** 0.27-0.70 | 46(7) | 0.10**(<0.001)** 0.08-0.12 | 0.05**(<0.001)** 0.02-0.08 |

* *Reference group is participants without impairment, adjusted for age (reference below 30 years old) and gender (reference female).* *95%CI: 95% confidence interval; aOR: odds ratio after adjusting for gender and age; statistically significant p-values are shown in bold.*
